# Heterogeneous expression of *CFTR* in insulin-secreting β-cells of the normal human islet

**DOI:** 10.1371/journal.pone.0242749

**Published:** 2020-12-02

**Authors:** Mauricio Di Fulvio, Marika Bogdani, Myrian Velasco, Timothy S. McMillen, Cecilia Ridaura, Lisa Kelly, Mohammed M. Almutairi, Shams Kursan, Abu A. Sajib, Marcia Hiriart, Lydia Aguilar-Bryan

**Affiliations:** 1 Department of Pharmacology and Toxicology, School of Medicine, Wright State University, Dayton, OH, United States of America; 2 Matrix Biology Program, Benaroya Research Institute, Seattle, WA, United States of America; 3 Departamento de Neurociencia Cognitiva, Instituto de Fisiología Celular, Universidad Nacional Autónoma de México, Mexico City, Mexico; 4 Pacific Northwest Research Institute, Seattle, WA, United States of America; 5 Departamento de Patología, Instituto Nacional de Pediatría, Mexico City, Mexico; 6 Department of Genetic Engineering and Biotechnology, University of Dhaka, Dhaka, Bangladesh; Centre de Recherche des Cordeliers, FRANCE

## Abstract

Cystic fibrosis (CF) is due to mutations in the CF-transmembrane conductance regulator (*CFTR*) and CF-related diabetes (CFRD) is its most common co-morbidity, affecting ~50% of all CF patients, significantly influencing pulmonary function and longevity. Yet, the complex pathogenesis of CFRD remains unclear. Two non-mutually exclusive underlying mechanisms have been proposed in CFRD: *i*) damage of the endocrine cells secondary to the severe exocrine pancreatic pathology and *ii*) intrinsic β-cell impairment of the secretory response in combination with other factors. The later has proven difficult to determine due to low expression of *CFTR* in β-cells, which results in the general perception that this Cl^−^channel does not participate in the modulation of insulin secretion or the development of CFRD. The objective of the present work is to demonstrate *CFTR* expression at the molecular and functional levels in insulin-secreting β-cells in normal human islets, where it seems to play a role. Towards this end, we have used immunofluorescence confocal and immunofluorescence microscopy, immunohistochemistry, RT-qPCR, Western blotting, pharmacology, electrophysiology and insulin secretory studies in normal human, rat and mouse islets. Our results demonstrate heterogeneous *CFTR* expression in human, mouse and rat β-cells and provide evidence that pharmacological inhibition of *CFTR* influences basal and stimulated insulin secretion in normal mouse islets but not in islets lacking this channel, despite being detected by electrophysiological means in ~30% of β-cells. Therefore, our results demonstrate a potential role for *CFTR* in the pancreatic β-cell secretory response suggesting that intrinsic β-cell dysfunction may also participate in the pathogenesis of CFRD.

## Introduction

As the treatment for patients with cystic fibrosis (CF) improved, they in turn became the population with the highest risk for developing age-dependent diabetes, the most common co-morbidity (50% in CF [1]), referred to as CF-related diabetes (CFRD). This is a highly relevant clinical issue because it worsens lung function and increases mortality rate [2]. CFRD is a complex hyperglycemic metabolic syndrome with features of type 1 and 2 diabetes mellitus that has been classified as *pancreatogenic* in origin (Type 3c) [3]. However, at least in infancy, built on our recent findings CFRD cannot be attributed to exocrine pancreatic pathology, but rather to a reduction in β-cell density and replication [4].

The direct role that *CFTR* plays in the insulin secretory response remain challenging due, at least in part, to two experimental limitations including: *i*) lack of animal models *fully* recapitulating the clinical picture present in human CF/CFRD [5], and *ii*) few reliable immunological [6] and pharmacological [7] tools to adequately identify and characterize channel expression and activity *in vitro*. The lack of tools is further exacerbated by the low and heterogeneous expression pattern of *CFTR* compared to the usual target tissues, *i*.*e*., lung, gut and exocrine pancreas [8–10], in the pancreatic islet; clearly adding important methodological obstacles for its detection and subsequent characterization. As a result, immunoreactive *CFTR* and/or its function have not been detected in the pancreatic islet by some [11,12] yet it has been by others [13–18]. Islet or β-cell *CFTR* mRNA expression has been recently interrogated [11], or not found [19] based on RNA-sequencing data. New results by White *et al*. suggest that *CFTR* is indeed expressed in a few β-cells in the adult human islet, but considered it too low and unlikely to play a role on β-cell function [12]. The inconsistency in the results from several laboratories could be explained, at least in part, by the fact that islet β-cells are of diverse morphology and functionally heterogeneous, which would make their detection difficult [20–22]. Hence, it remains possible that *CFTR* may be expressed in a subpopulation of β-cells and therefore, play a role in their secretory response.

*CFTR*, like any other Cl^−^channel in the islet, has the potential to regulate the insulin secretory response. Indeed, as we have recently reviewed in detail [23], intracellular Cl^−^ions tend to electrogenically exit from insulin-secreting β-cells through Cl^−^channels thus contributing to plasma membrane depolarization [24] and therefore insulin secretion [25]. The objective of the present study was to detect *CFTR* expression at the molecular and functional levels in the human and rodent pancreatic islet whilst testing the hypothesis that *CFTR* expression occurs in a heterogeneous pattern in a subpopulation of β-cells. Our results confirm and extend previous reports on the expression, localization and function of *CFTR* in human and rodent β-cells and for the first time, we present electrophysiological data suggesting that ~30% of all β-cells tested express functional *CFTR*.

## Materials and methods

### Materials

Platinum *Pfx* thermostable DNA polymerase, RNaseOUT, SuperScript-III reverse transcriptase, random hexamers, and tissue culture media were from Invitrogen (Carlsbad, CA); dNTPs and molecular biology grade chemicals were from Affymetrix (Cleveland, OH); custom PCR primers were from Integrated DNA Technologies (Coralville, IA). Tissue culture supplements and general chemicals were from Sigma-Aldrich Co. (St. Louis, MO). Culture media low in glucose (5.5mM) was from Hyclone-GE (Logan, UT). Microscopy materials were from EMS (Hatfield, PA) and ThermoFisher Sci. (Waltham, MA). Collagenase type IV from *Clostridium histolyticum* (≥160U/mg) was from Worthington (Lakewood, NJ). *CFTR* inh-172 and the adenylyl cyclase activator forskolin where from Tocris and R&D (Minneapolis, MN).

### Human tissue and islet donors

**Fig 1:**
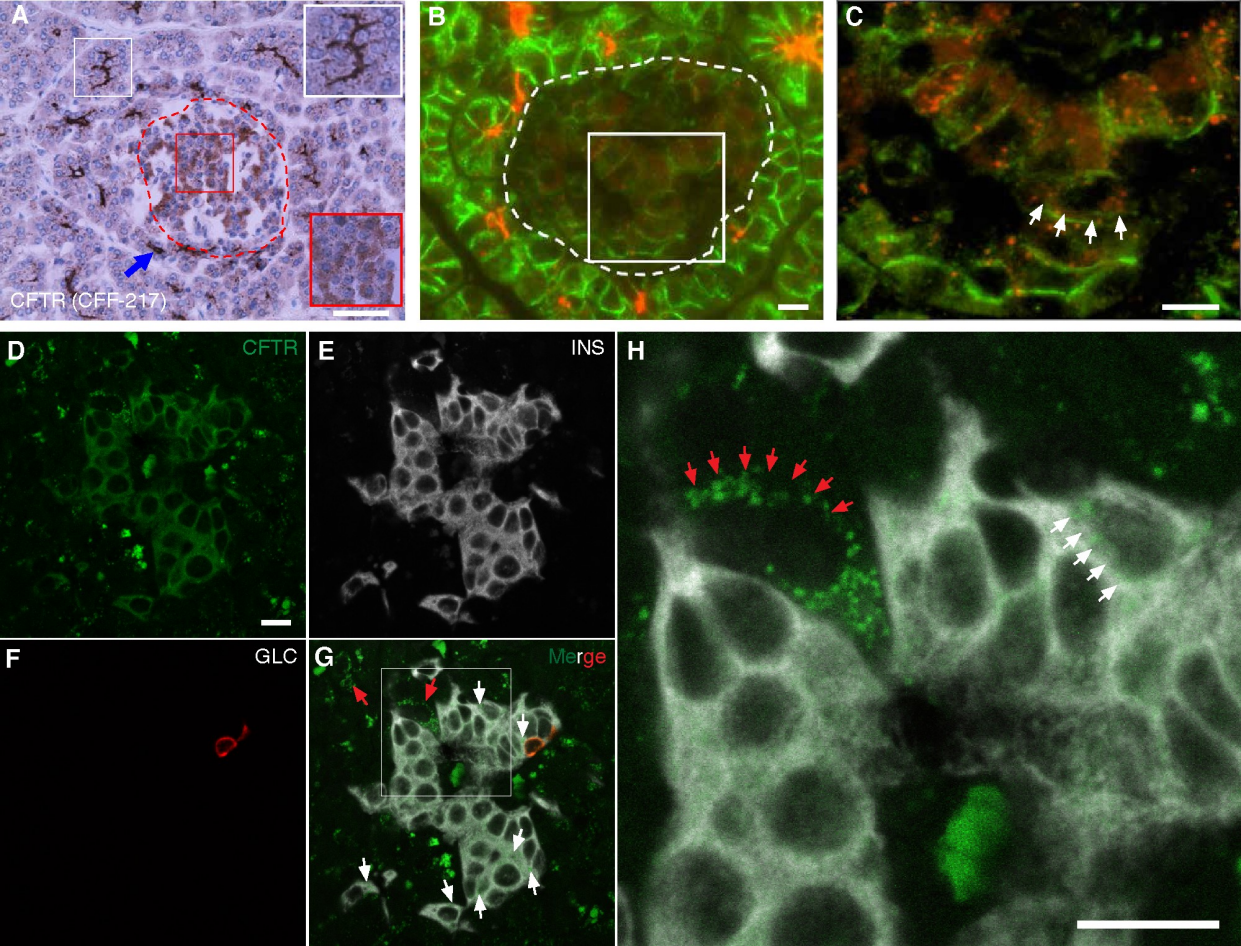
*CFTR* is expressed in human islets. **A-C.** Immunohistochemistry (A) and immunofluorescence (B-C) images of human pancreas (normal tissue from an 11-month-old control) immunostained by using CFF-217 antibody (Table 1). The islet shown in A, encircled by a dashed red trace, contains endocrine cells stained by the antibody, which is shown in the magnified red square in the right bottom corner of the figure. *CFTR*-positive pancreatic duct cells are shown within the white squares. Shown in B is *CFTR* immunoreactivity in human exocrine and endocrine cells. The edges of all cells were immunolabeled by using E-cadherin antibodies (Table 1). The islet is encircled by a dashed trace and the square represents the area magnified and shown in C. **D-H.** *In situ* hybridization of normal human pancreas tissue by using fluorophore-labeled RNA probes directed against *CFTR* (green, D), insulin (*INS*, white, E) and glucagon (*GLC*, red, F) transcripts. Red and white arrows in H, a magnification of G, indicate specific *CFTR* labeling on acinar and endocrine cells, respectively. Bars in A and B, C, D, and in H correspond to 50μm and 10μm, respectively.

**Fig 2:**
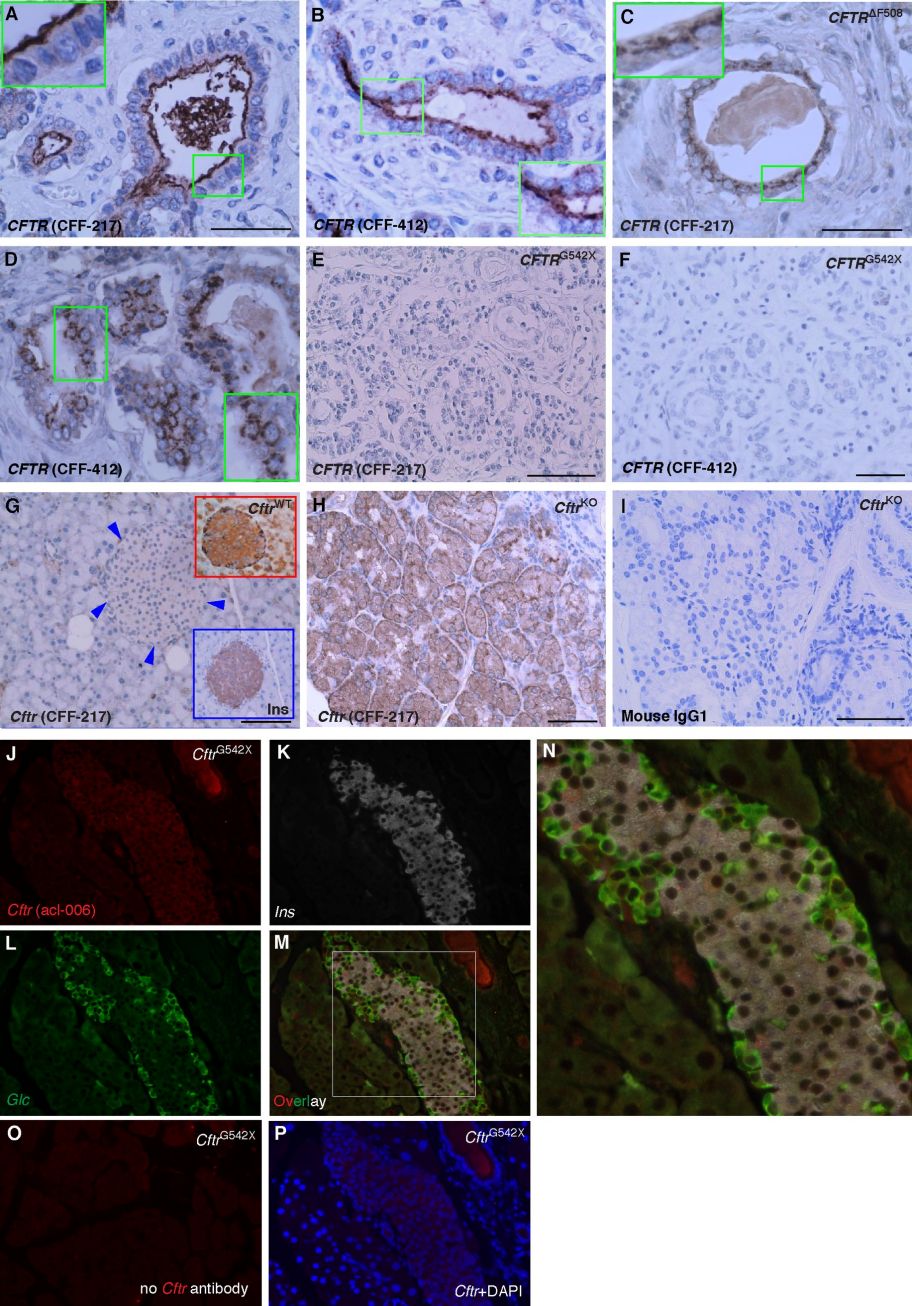
*CFTR* antibody validation. **A-D.** Shown are microscopy images of human pancreas from a normal donor (A, B, 11-month-old control) or homozygous for the *CFTR* mutation F508del (C, D, 6-month-old CF patient) immunostained using CFF-217 (A, C) or CFF 412 (B, D). Note the expected apical and intracellular immunolabeling of *CFTR* in normal and mutant pancreas, respectively. The insets indicate higher magnification. **E, F.** Microscopy images of human pancreas from a 1 yr. old CF patient homozygous for *CFTR*^G542X^ using the antibodies CFF-217 (E) and CFF-412 (F). Shown is absence of immunostaining with both antibodies. **G-I.** Microscopy images of pancreas (G) and intestine (H) of *Cftr*^KO^ mice immunolabeled using CFF-217. Shown are the expected patterns of immunostaining for specific antibodies. For comparison, shown are magnified images of *Cftr*^WT^ pancreas islets using *CFTR* and insulin antibodies (top and bottom right corners in G, respectively). **I.** Omission of the *CFTR* antibody did not generate immunostaining in the intestine. Bars in A-I represents 50μm. **J-L.** Pancreatic tissue from *Cftr*^G542X^ mice co-immunolabeled by using the indicated mouse *CFTR*-specific antibody acl-006 (J), insulin (K, *Ins*) and glucagon (L, *Glc*). **M-N.** Overlay image of J-L and magnified squared area shown in N. **O-P.** Control images obtained from *Cftr*^G542X^ mice in the absence of primary (O) or secondary antibodies (P). DAPI was used to counterstain nuclei in P.

Normal adult and juvenile human pancreatic tissue for IHC was obtained from Dr. Cecilia Ridaura (Departamento de Patología, Instituto Nacional de Pediatría, Mexico City) and from the Network for Pancreatic Organ Donors (nPOD, JDRF). At the beginning of this project, we obtained 36 tissue samples; 20 CF cases, out of which 16 were less than one year of age and 4 between 20 and 40 years and 11 control samples from patients younger than 24 months of age and 5 adults, who died of non-pancreatic pathology [4]. Tissue sections from a large subset of this group was used to test 21 antibodies against *CFTR* (see Table 1 and S1–S4 Figs), of those, sections from 6/12 and 12/12 CF patients (homozygous for the *CFTR* mutations F508del and G542X, of Mestizo origin), as well as an 11/12 control case are presented in Figs 1 and 2. All the experiments with these de-identified archival autopsy tissues obtained from deceased patients were carried out with the approval of the Institutional Review Board of the Instituto Nacional de Pediatría. The excellent quality of these tissues was incontestable [4]. Primary human islets from normal donors were obtained from Prodo Laboratories (Aliso Viejo, CA). Detail demographics are presented in Table 2. Upon arrival, primary islets were allowed to recover 24-48hs in PIM(R) media supplemented with PIM (ABS, G & 3X) as per provider instructions and subsequently used for functional, molecular and secretory studies.

**Table 1:** List of primary antibodies raised against the indicated antigens are identified by their clone/catalogue number from vendors/suppliers.

Antibody	Supplier	Dilution	Tested application	Comments
Cftr (CFF-432)	Cystic Fibrosis Foundation (CFF)	1:250	IHC/WB	Specific, tested against KO tissues, not suitable for WB
Cftr (CFF-217)	CFF	1:250	IHC/WB/IF	Specific, tested against KO tissues, not suitable for WB
Cftr (CFF-412)	CFF	1:250	IHC/WB	Specific, tested against KO tissues, not suitable for WB
Cftr (CFF-596)	CFF	1:100	WB	Not specific, not suitable for WB
Cftr (10B6.2)	CFF	1:100	WB	Specific, not suitable for WB
Cftr (CFF-570)	CFF	1:100	IHC/WB	Specific, not suitable for WB
Cftr (CFF-770)	CFF	1:100	IHC/WB	Not suitable for IHC/WB
Cftr (CFF-154)	CFF	1:50	IHC	No signal
Cftr (CFF-660)	CFF	1:100	IHC	Not specific, high background
Cftr (CFF-450)	CFF	1:50	IHC	Strong staining in islets and weak in ducts
Cftr (MA1-935)	Thermo Scientific	1:400	IHC	Stains islets and ducts
Cftr (Ab4067)	Abcam	1:100	IHC	No signal in human pancreas, weak in mouse pancreas
Cftr (Ab-3 L1B4)	Neomarkers	1:50	IHC	No signal
Cftr (acl-006)	Alomone	1:250	IF	Specific
Cftr (24–1)	R&D	1:250	WB	Specific, not suitable for IF
Cftr (MrPink)	CFTR Folding Consortium	1:250	IF	Specific
Cftr (3G11)	CFTR Folding Consortium	1:250	IF	Specific
Cftr (LS-B5115)	Ls-Bio	1:200	IF	No signal
Cftr (NB300-511)	Novus Biologicals	1:100	IF	Very weak signal
Cftr (20738-1-ap)	Proteintech	1:100	IF	No signal
Cftr (Mab3484)	Millipore	1:250	IHC/IF	Not suitable for IF
Insulin (273A-16)	Cell Marque	1:500	IF/IHC	Widely used, considered specific
Glucagon (K79bB10)	Abcam	1:500	IF/IHC	Widely used, considered specific
Glucagon (259A-15)	Cell Marque	1:500	IF/IHC	Widely used, considered specific
Somatostatin (ab30788)	Abcam	1:250	IF/IHC	Widely used, considered specific
Synaptophysin (ab8049)	Abcam	1:200	IF/IHC	Widely used, considered specific
PP (ab77192)	Abcam	1:250	IF/IHC	Widely used, considered specific
E-cadherin (ab6528)	Abcam	1:100	IF	Widely used
Actin (JLA20)	DHSB	1:5000	WB	Specific

Dilutions and applications used are indicated together with comments regarding their specificity/reliability/suitability. *Note*: suitability refers to the ability of the antibody to detect low expression of the antigen in the pancreatic islet. *CFTR* antibodies were tested in pancreatic tissue slides and/or protein extracts, as indicated.

**Table 2:** Human islet donor demographics.

Age (years)	Sex	Ethnicity	Height (cm)	Weight (kg)	BMI	HbA1c (%)	Cause of Death	Purity (%)	Viability (%)	Secretion	Gene expression
48	M	White	172.0	81.7	26.3	5.1	Stroke	90	95	✓	
61	F	White	162.5	66.3	24.5	5.1	Stroke	95	95		✓
52	F	White	170.1	73.5	25.6	4.5	Stroke	95	95		✓
38	M	Latino	175.3	86.5	28.0	5.9	Head trauma	90	95	✓	
46	F	White	170.0	55.8	19.0	5.8	Stroke	90	95	✓	
31	M	White	175.2	72.2	22.0	5.5	Anoxic event	95	95	✓	
33	F	White	172.7	90.8	30.3	4.9	Head trauma	85	95		✓
49	M	White	170.2	86.3	30.0	5.5	Anoxic event	90	95	✓	
35	M	African-American	170.0	85.0	30.2	5.4	Anoxic event	90	95	✓	
33	F	White	172.7	90.8	30.3	4.9	Head trauma	85	95	✓	
61	F	Latino	149.9	64.9	28.9	5.6	Head trauma	90	95	✓	
54	M	White	180.3	72.6	22.3	5.8	Stroke	90	95	✓	
35	F	White	160.0	62.7	24.0	4.8	Anoxic event	90	95	✓	
47	M	White	160.0	71.0	28.1	5.8	Head trauma	85	95		✓
43	F	Latino	152.4	68.1	30.0	5.5	Head trauma	95	95		✓
46	M	Latino	167.6	93.0	33.0	5.4	Anoxic event	90	95		✓
30	F	White	170.2	54.5	18.0	4.4	Anoxic event	90	95		✓
40	M	Latino	175.3	78.1	25.0	5.3	Head trauma	95	95		✓
49	F	White	155.0	81.3	33.0	5.9	Stroke	90	95		✓
52	F	African-American	170.2	69.5	23.0	4.8	Stroke	90	95		✓
57	F	White	167.6	59.9	21.4	5.8	Stroke	95	95		✓
63	F	White	152.0	52.7	27.0	5.8	Stroke	85	95	✓	
66	M	White	170.2	79.0	27.0	4.7	Stroke	95	95	✓	
50	M	Latino	160.0	84.0	32.6	6.0	Anoxic event	90	95	✓	

### Mouse pancreas collection and processing for immunomicroscopy

Fixed pancreas tissues from wild-type (WT) mice of the C57BL/6J genetic background and homozygous for the *Cftr*^G542X^ allele were kindly provided by Dr. Mitch Drumm (Case Western Reserve University). Tissues from mice homozygous for the *Cftr* mutation S489X and expressing the human *CFTR* transgene in the intestine [*Cftr*^*tm1Unc*^ Tg(FABPCFTR)1Jaw/J, Stock No: 002364, Jackson Labs], referred to as *Cftr*^KO^ mice throughout the text were obtained and processed as described [26] with some modifications. Briefly, deeply anesthetized mice (Euthasol^®^, *ip* 150mg/kg) were transcardially perfused with ~20ml ice-cold 1xPBS (0.1mM, pH7.4) containing 1000U/ml of heparin (Meitheal Pharmaceuticals, IL) at a rate of 1.6ml/min with the help of a peristaltic minipump (Model EP-1 Econo pump, Bio-Rad) and then perfused with freshly made ice-cold 4% paraformaldehyde (PFA) fixative. After mice were euthanized by perfusion with PFA, pancreatic tissue was collected, placed at 4°C overnight in 4% PFA, washed in PBS and post-fixed for additional 24hs in 4%PFA containing 30% sucrose. Post-fixed tissues were transferred to 30% sucrose in PBS and collected after tissues sunk at the bottom of the tube. Tissues were paraffin-embedded and sectioned at 5 μm (AML Laboratories, Inc., Baltimore, MD). For immunolabeling protocols, tissue slides were deparaffinized in xylene and re-hydrated in ethanol solutions of decreasing concentrations (100%-0%). Antigens were retrieved in sodium citrate buffer (10 mM) at 100°C for 30 min, tissue slides permeabilized in 4% PFA containing 0.3% Triton X100, blocked in 3% host serum solution and incubated with primary antibodies as indicated in the next section.

### Immunofluorescence/confocal microscopy, immunohistochemistry and *in situ* hybridization

The twenty-one primary antibodies used in these experiments and their sources are listed in Table 1. Tissue sections from mouse or human pancreas were immunolabeled with different dilutions of the primary antibodies at 4°C overnight with gentle rocking followed by 2hs incubation with appropriate fluorescently-labeled secondary antibodies [Cy3-, AlexaFluor (AF488)- and DyLight405-conjugated (Jackson Immunoresearch, West Grove, PA). Slides were allowed to dry at room temperature before adding mounting media containing DAPI (Vector Laboratories, Burlingame, CA) and cover the sections with acetone-washed coverslips. Labeled slides were immediately viewed using an Olympus Epi Fluorescence Spot microscope equipped with RT color camera. Digital images were obtained using a Diagnostics Instrument Spot 6 digital camera (Spot Imaging Solutions, Sterling Heights, MI). For immunohistochemistry (IHC) experiments, tissue sections were incubated overnight with primary antibodies. Sections were washed in 1xPBS and then incubated with biotinylated- or fluorescent-labeled secondary antibodies for 1h at room temperature. The peroxidase (HRP) reaction was developed by incubating sections in 0.3% H_2_O_2_ and 0.15% diaminobenzidine tetrachloride (Sigma, St. Louis, MO). For *CFTR*, detection the antibodies we used were all validation against *CFTR* null tissues following identical conditions (see Figs 2C, 2E, 2F, 2H–2P and 4A). Classic negative controls were also performed by omitting primary antibodies (Fig 2O) or by staining with isotype-matched antibodies (Fig 2I). Some of the antibodies not suitable for immunohistochemistry experiments are shown in S3 Fig. Neither of these two approaches generated staining. *In situ* RNA hybridization was performed using RNAscope technology (ACD, Advanced Cell Diagnostics, CA). Normal human pancreas sections provided by ACD were permeabilized and hybridized with combinations of mRNA probes specific for human *CFTR*, insulin (*INS*) and glucagon (*GLC*) according to the manufacturer’s instructions. A multiplex fluorescent kit was used to detect mRNA signals, which were analyzed by epi-fluorescence and confocal microscopy. High-resolution confocal images were taken by using the FV1000 Confocal Microscope (Olympus, PA, USA). When DyLight405-conjugated antibodies were used to visualize insulin-positive β-cells, images were taken in gray-scale instead of blue color to increase contrast against red- and green-stained antigens.

### Mice and rats

*Cftr*^WT^, *Cftr*^KO^ [*i*.*e*., *Cftr*^*tm1Unc*^Tg(FABPCFTR)1Jaw/J] and C57BL/6J mice (Jackson Laboratories) were housed under controlled conditions at the Cell Function Analysis Core of the Diabetes Research Center (DRC, University of Washington, Seattle, WA). Male and female mice (8–10 weeks old) were used. The Animal Care Committee of the University of Washington approved all methods involving mice. Male Wistar rats (12 young adults, 250-280g) were obtained from the local animal facility and housed at the Instituto de Fisiología Celular, Universidad Nacional Autónoma de México. Rats were kept in a 12:12h light-dark cycle under standard laboratory conditions including and fed with standard rat chow (Laboratory rodent diet 5001, LabDiet St Louis, MO, USA) and tap water. The Animal Care Committee of the Instituto de Fisiología Celular, Universidad Nacional Autónoma de México approved all methods involving rats. Animal care (mice and rats) was performed according to the International Guiding Principles for Biomedical Research Involving Animals, Council for International Organizations of Medical Sciences, 2010.

### Primary islet and tissue isolation

Mouse islets for secretory studies were isolated from by the DRC Cell Function Analysis Core as previously described [27]. Mice and rat pancreas were obtained from deeply anesthetized animals (Euthasol^®^ *ip* 150mg/kg or 40mg/Kg sodium pentobarbital for mice and rats, respectively) by the terminal collagenase digestion method as described [28]. After surgical removal of the collagenase-digested pancreas, mice and rats were immediately euthanized. Tissues for gene expression analysis were surgically removed from deeply anesthetized mice (Euthasol^®^ *ip* 150mg/kg) and immediately processed.

### Insulin secretion

Primary islets were handpicked into individual wells of 12-well plates with mesh inserts [15 islet equivalents (iEq)/well] containing KRBH (in mM: 118.5 NaCl, 2.5 CaCl_2_, 1.2 KH_2_PO_4_, 4.7 KCl, 25 NaHCO_3_, 1.2 MgSO_4_, 10 HEPES and 0.1% BSA pH 7.4) plus 5.5mM glucose. The mesh inserts containing islets were transferred to new wells containing KRBH+5.5mM glucose and incubated at 37°C (5% CO_2_) for 30 minutes. This wash step was repeated once more. The islets were then transferred into their respective experimental wells containing KRBH + 5.5mM glucose plus vehicle (DMSO) or drugs for 2h at 37°C (5% CO_2_). Islets were transferred into new wells containing KRBH + 12.5mM glucose plus vehicle or drugs, incubated 2h at 37°C (5% CO_2_) and transferred to new wells containing acidified ethanol. The KRBH from experimental wells was frozen at –20°C until analysis. Insulin secreted into the media or contained in cells was estimated by using ELISA (#10-1247-01, Mercodia, Salem, NC). Results are expressed as the % ratio between secreted insulin and the sum of secreted and islet/cell insulin content. When assayed at DRC, the insulin secretory response (ISR) was determined statically with multiple conditions, as described previously [29]. Briefly, primary islets were handpicked into a petri dish containing KRBH+5.5mM glucose and incubated at 37°C (5% CO_2_) for 90min. Subsequently, islets were picked into 96-well plates containing desired amounts of glucose and drugs, as indicated, and incubated for additional 60min. At the end of this period, supernatant was assayed for insulin by radioimmunoassay (Linco Research Inc., Billerica, MA). Results are expressed as ISR per min, per 100iEq. Single rat islet cells were obtained by incubating handpicked islets in a shaker bath at 37°C in Ca^2+^-free spinner solution containing 10mM glucose, 0.5% BSA and 0.01% trypsin, followed by mechanical disruption. Single cells were cultured overnight on poly-L-lysine pre-coated glass coverslips and incubated in RPMI-1640 (11.6mM glucose) supplemented with 10% FCS, 200U/ml penicillin G, 200 μg/ml streptomycin and 0.5 μg/ml amphotericin-B for recover from dissociation. Mouse MIN6 β-cells for secretory studies were kept, cultured and assayed as previously described [30]. Basal or glucose-stimulated insulin secretion (GSIS) was determined in these cells two hours after incubation in the presence of basal (5.5mM) or stimulatory glucose concentrations (12.5mM) by ELISA (EZRMI-13K, EMD Millipore, Billerica, MA) in accordance with the manufacturer’s directions.

### Electrophysiology

Whole-cell configuration of patch-clamp technique was used on isolated primary rat β-cells. All the experiments were done at 22°C. Ionic currents were recorded with a data acquisition system (DigiData 1322A) and Axopatch 200A amplifier (Axon Instruments, Foster City, CA USA). Patch pipettes from capillary tubes KIMAX-51 (Kimble Glass, Vineland NJ) were coated with dental wax and pulled with micropipette puller (P-97, Sutter Instruments Co., USA) to a resistance of 2–4 MΩ after being filled with intracellular solution. The cells were bathed with an external solution (in mM): 118 NaCl, 20 tetra-ethyl-ammonium-chloride, 5.6 KCl, 2.6 CaCl_2_, 1.2 MgCl_2_ and 5 HEPES with 5.5 or 12.5 D-Glucose pH 7.4, supplemented with 10μM forskolin and 5μM Inh172. The pipettes were filled with solution (in mM): 125 CsOH, 125 glutamate, 10 CsCl, 10 NaCl, 1 MgCl_2_, 3 MgATP, 4 EGTA, 5 HEPES pH 7.2. When the Giga seal was performed cells with capacitances between 5-12pF were considered as β-cells [28,31,32]. The whole-cell current was obtained by depolarizing test pulses from –100 to +100mV, at 10mV increments, from a holding potential of –80mV. The command voltages and analysis of currents were done with pClamp v9 software.

### Extraction of total RNA and RT-PCR

Total RNA was obtained from freshly isolated mouse as indicated above and human islets or β-cell lines using the RNeasy minikit (Qiagen, Valencia, CA). These RNA samples or purified total RNA from human islets (kindly provided by Dr. Patrick MacDonald, University of Alberta, Canada) were quantified by Nanodrop and used in RT-PCR/qPCR experiments as described [30]. The sets of primers used to amplify transcripts of interest are indicated in Table 3. RT-PCR amplicons were sequenced (Beckman Genomics, Beverly, MA) and aligned *in silico* using Geneious Suite R10 (Biomatters Ltd., New Zealand) against relevant sequences of reference (*RefSeq*). Thermal conditions used for PCR were previously described [30]. RT-PCR products were separated on 2% agarose gels stained with ethidium bromide and directly photographed by using the ChemiDoc™ MP Imaging System with Image Lab™ Software (Bio-Rad, Berkeley, CA). The digital images were inverted for clarity and cropped to exclude gel edges or empty/non-relevant lanes.

**Table 3:** List of primer-sets used in RT-PCR experiments named after the target transcript followed by numbers indicating amplicon sizes in base pairs (bp).

Primer set	Sense	Antisense	RefSeq	Species
Cftr-509	GTCATTCGACGAGTTCTAAAACAAG	CAAGAGTATATCCACAGGTATTGTCC	NM_021050	mouse
Cftr-511	CTAGTAGTCTTTATTTTACTGAGGGCC	CTAAAACGTCAGATGATCCTTCTCTAG		
Cftr-567	ATGTCGAGTCCAACCTGAATTTAG	CGGCTTGACAACTTTAAAGTCTTC		
CFTR-457	CATTCTGTTCTCAGTTTTCCTGGATTATGC	CTAAAGTCTGGCTGTAGATTTTGGAGTTCT	NM_000492	human
CFTR-547	CTCAAGAAACTGGCTTGGAAATAAGTGAAG	ATGAAGTCAAATATGGTAAGAGGCAGAAG		
CFTR-583	AGGGAGAATGATGATGAAGTACAGAGATCA	GAGAAATTACTGAAGAAGAGGCTGTCATC		
CFTR-594	CTGAATTTACATACTGCCAACTGGTTCTTG	CAAAGTTATTGAATCCCAAGACACACCATC		
Krt19-544	CCTGAAGAAGAACCATGAGGAGGA	CAGATTGTTGTAGTGGGCTTCCTG	NM_008471	mouse
GAPDH-555	GTGAAGGTCGGAGTCAACGGATTT	CACAGTCTTCTGGGTGGCAGTGAT	NM_002046	human

Primer sequences were designed according to mouse, rat or human cDNAs and/or *RefSeq* nucleotide sequences of reference, as indicated.

### Western blotting

Proteins from tissues and cell lines were obtained as formerly described [30]. Protein extracts from human islets were freshly obtained either from Dr. Patrick MacDonald or after sonication of freshly isolated primary human (Prodo Labs, Table 2)/mouse islets on ice by using RIPA buffer (Teknova, Hollister, CA) supplemented with protease/phosphatase inhibitor cocktails (ThermoFisher, Sci.). Up to 75μg of total proteins were loaded onto pre-casted 4–20% Tris-HEPES protein gels (Thermo Scientific-Pierce) or Bolt 4–12% Bis-Tris Plus gels (Invitrogen/ThermoFisher Sci.), run under denaturing conditions and electro-transferred onto PDVF membranes at 4°C by using the Mini Trans-Blot Electrophoretic Transfer Cell (Bio-Rad) or the iBlot-2 Gel Transfer Device (Invitrogen). After blocking overnight with 4% BSA, membranes were incubated with suitable primary antibodies (overnight at 4°C) and developed with HRP-conjugated secondary ones (2h at room temperature). We used ChemiDoc™ MP Imaging System with Image Lab™ Software (Bio-Rad, Berkeley, CA) to detect antigen-antibody reactions. High-resolution gray-scale digital images taken by using this imaging system were cropped to exclude irrelevant lanes and used without further modification. Original blots used to construct Figures are shown in S4 Fig.

### Statistics

Data has been analyzed by using Prism v5 (GraphPad Software, San Diego, CA) and pClamp v9 Electrophysiology Data Acquisition and Analysis Software (Molecular Devices, LLC. San José, CA). Results are represented as mean values ± SEM, with the number of individual replicates (*n*). A *p* value ≤0.05 was considered significant and was obtained by using one-way or two-way analyses of variance (ANOVA), as appropriate, followed by the Tukey-Kramer *post-hoc* test.

## Results

### *CFTR* is expressed in endocrine cells in the human islet

*CFTR* protein and mRNA expression were determined in the human pancreatic islet by using immunomicroscopy and *in situ* hybridization, respectively. We have evaluated twenty-one different antibodies against *CFTR*, of which only a handful were specific or suitable for immunomicroscopy experiments (Table 1 and S1–S3 Figs). As shown in Fig 1A–1C, *CFTR* antibodies CFF-217 and CFF-412 detect *CFTR* in a fraction of the normal human islet endocrine cells with the expected intense staining and apical localization of the pancreatic duct cells (*inset* Fig 1A). Similar results are shown in S2 Fig using *CFTR* antibodies CFF-432, -412 and -570. Corroborating the concept that *CFTR* is expressed in the human islet, the channel was sought at the mRNA level by using *in situ* hybridization. As shown in Fig 1D–1H, very low expression of *CFTR* transcripts is detected as a small punctate in some but not all β-cells in the human islet. Importantly, the *CFTR* antibodies used in our experiments gave the predicted predominantly apical as well as cytoplasmic immunostaining pattern in pancreatic sections from control patients (Figs 2A, S1 and S2), or only cytoplasmic in the case of patients homozygous for the mutation F508del (ΔF508, Fig 2B) but did not generate an immunological signal in pancreas sections from a CF patient homozygous for the truncating *CFTR* mutation G542X (Fig 2C and 2D) leading to protein absence, or mice lacking *Cftr* expression (Fig 2E and 2F).

**Fig 3:**
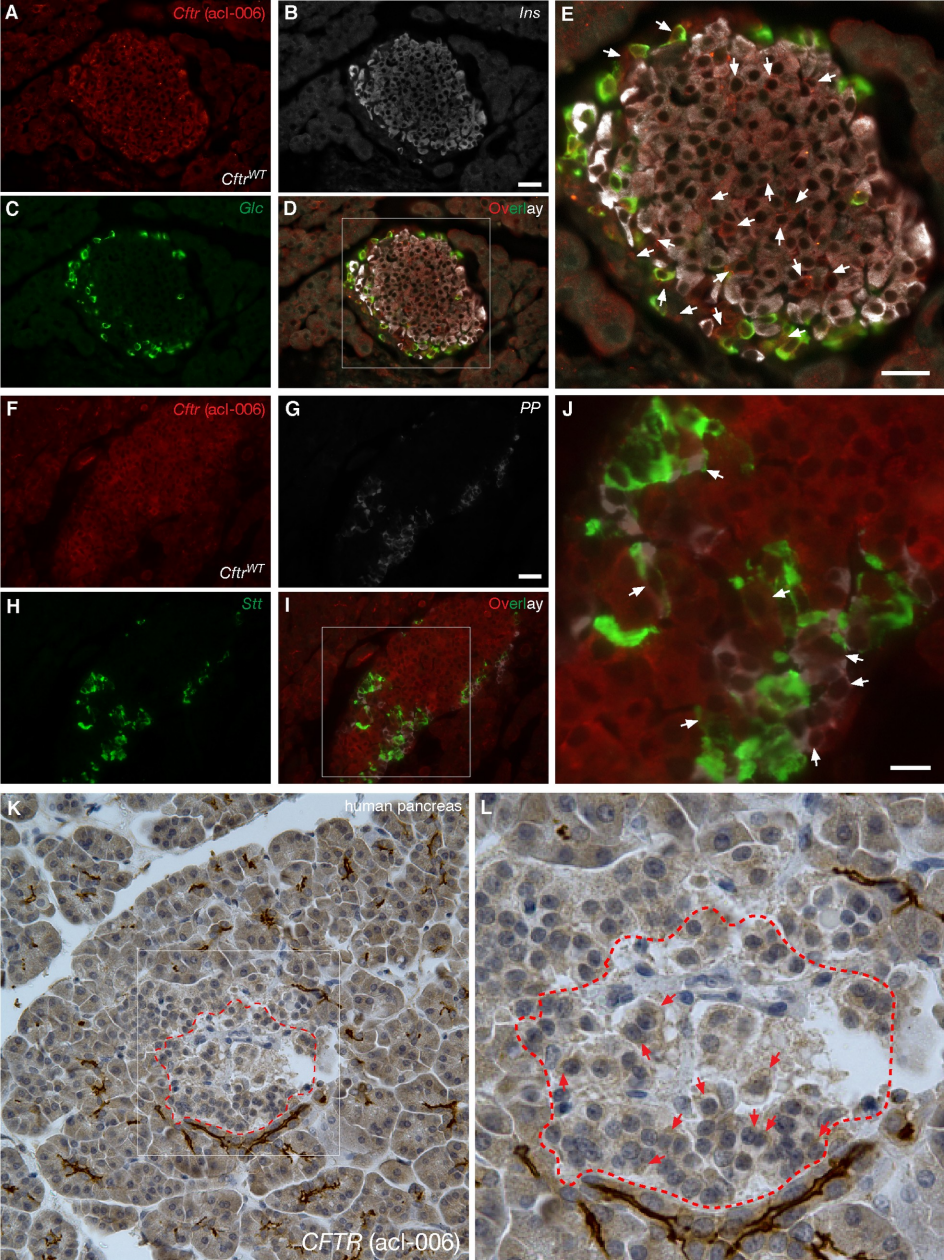
*Cftr* is expressed in endocrine cells of the mouse islet. **A-J.** Immunofluorescence images of WT mouse (*Cftr*^WT^) pancreas immunostained with the *Cftr* antibody acl-006 [(Table 1), A and F] in combination with antibodies against insulin (*Ins*, B), glucagon (*Glc*, C), somatostatin (*Stt*, H) and pancreatic polypeptide (*PP*, G). Magnified overlay images of D and I are shown in E and J, respectively, wherein *Cftr* immunoreactivity in α-, β-, δ- and PP-cells of the mouse islet is indicated by arrows. **K-L.** Immunohistochemistry images of normal human pancreas immunostained with acl-006. The islet encircled by a dashed red trace in K is magnified in L to show *CFTR* immunoreactivity. Bars in A-G represent 25μm.

**Fig 4:**
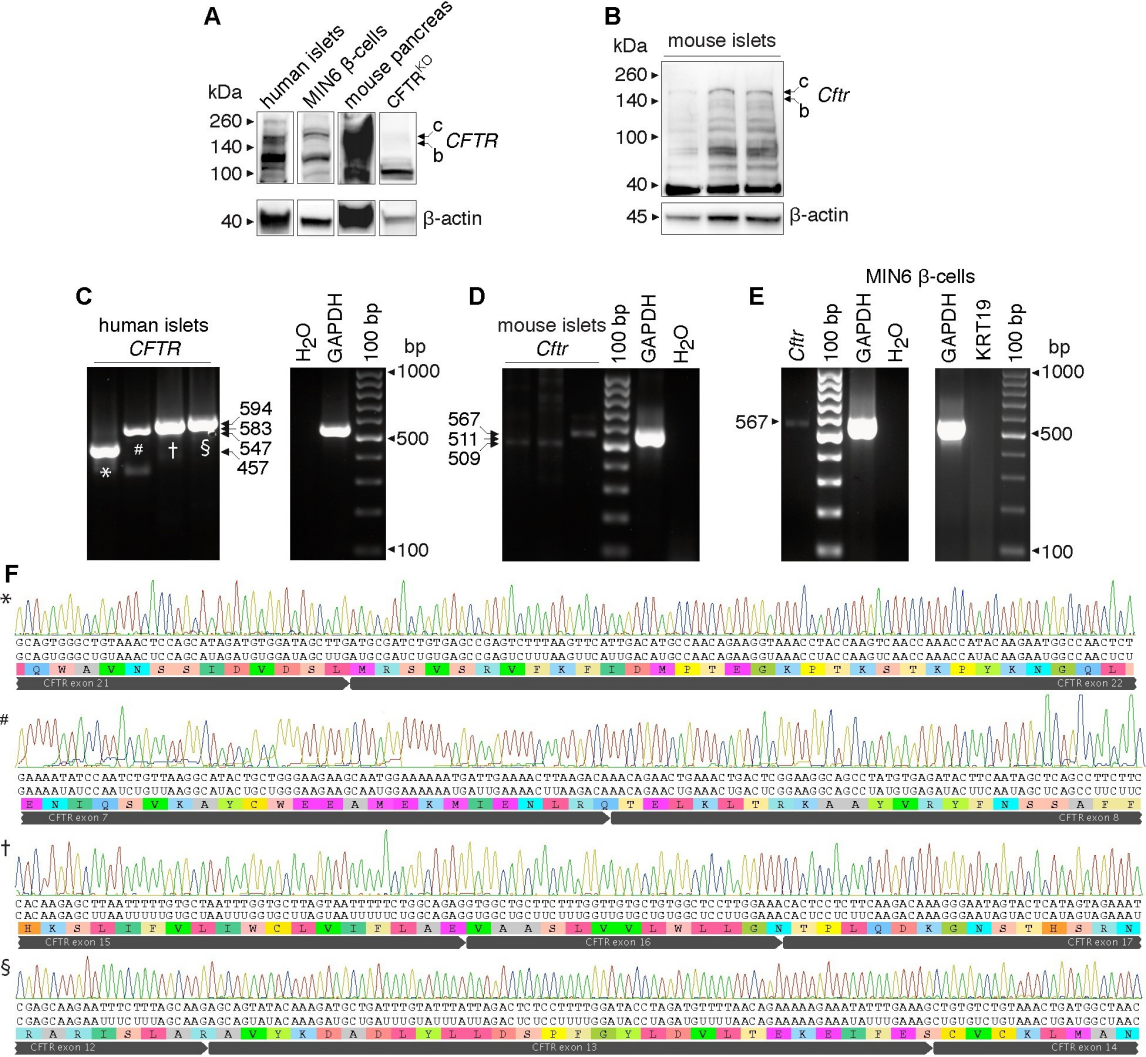
*CFTR* expression in human and mouse islets and in MIN6 β-cells. **A, B.** Immunoblots of protein extracts obtained from purified human islets, the β-cell line MIN6 and mouse pancreas as positive control (A) and primary mouse islets (B). Bands of expected size are shown for human/mouse *Cftr* (C band: ~170kDa, B band: ~150kDa) detected using *Cftr* antibody 24–1. Actin was used as loading control. **C-E.** Representative original RT-PCR experiments showing *Cftr* transcripts of expected sizes (Table 3) amplified from total RNA obtained from primary human (*n* = 5 donors), mouse islets (*n* = 3 preparations) (C and D, respectively) and MIN6 β-cells (*n* = 4 preparations) (E). **F.** Partial sequence chromatograms of purified human *CFTR* amplicons, as indicated in C.

To further ratify and extend the previous observations, glucagon, insulin, somatostatin, pancreatic polypeptide (PP) and *Cftr* immunoreactivity were also sought in normal rodent islets. *CFTR* was detected by using the *Cftr* antibody acl-006 (Table 1), also validated against pancreatic sections from mouse islets homozygous for *Cftr*^G542X^ allele (Figs 2H and 3). As shown in Fig 3, immunoreactive *Cftr* is detected in some but not all *Cftr*^WT^ mouse α-, β- (Fig 3A–3E), δ- and PP-cells (Fig 3F–3J). In a similar fashion, Fig 3K and 3L show the expected immunolabeling of the endocrine and exocrine components of the human pancreas. To cross-validate these results, *Cftr* expression was demonstrated at the protein (Fig 4A and 4B) and mRNA (Fig 4C and, 4D) levels in human islets ~95% free of exocrine cells (Table 2), mice primary islets as well as in the mouse β-cell line MIN6 (Fig 4E), which is clearly exempt of exocrine contamination. Of note, the molecular identity of the *CFTR* mRNAs amplified from human islets was verified by direct DNA sequencing (Fig 4F). When taken together, these results confirm and broaden previous observations that *CFTR* is in fact expressed in the human and mouse islet, albeit at a low level and in a small percentage of endocrine cells.

### *CFTR* modulates insulin secretion

**Fig 5:**
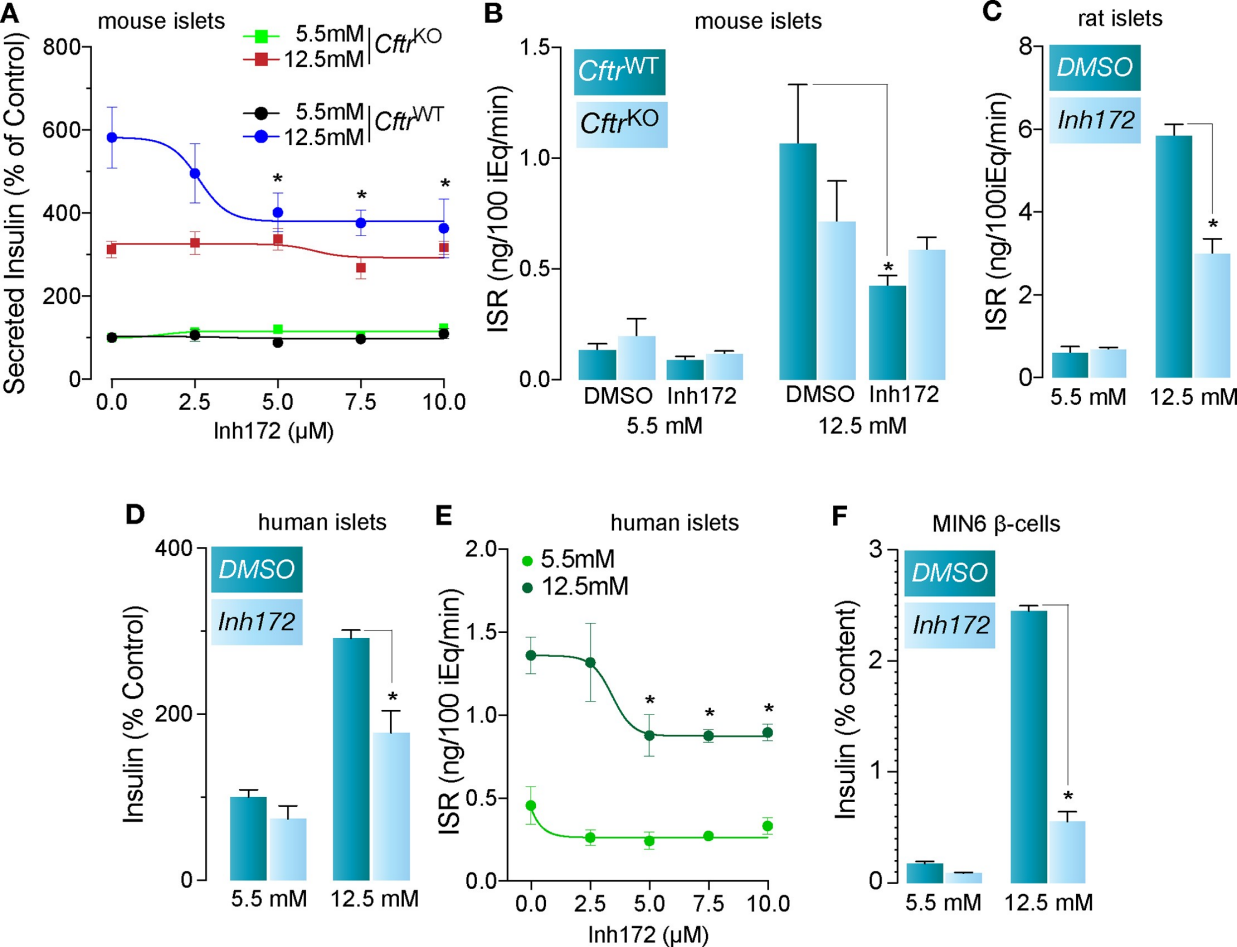
*Cftr* contributes to the insulin secretory response in mouse, rat and human islets and the MIN6 β-cell line. **A.** Proof of specificity for CFTRinh-172 (0–10μM) on the secretory response using islets obtained from transgenic mice lacking *Cftr* (*Cftr*^KO^) and *Cftr*^WT^ mice, in response to 5.5mM and 12.5mM glucose (*n* = 8, *p<0.05). **B, C.** Effect of 5μM CFTRinh-172 on insulin secretion of mouse *Cftr*^KO^ and *Cftr*^WT^ islets (B, *n* = 5, *p<0.05) and rat islets (C, *n* = 3, *p<0.05) in response to 5.5mM and 12.5mM glucose. **D.** Basal (5.5mM glucose) and stimulated (12.5mM glucose) insulin secretory response of freshly isolated primary human islets in the presence of vehicle (DMSO) or 5μM CFTRinh-172 (*n* = 5 donors, *p<0.05). **E.** Dose-response curve of basal (5.5mM glucose) and stimulated (12.5mM glucose) insulin secretion from human islets (*n* = 4 donors, *p<0.05) treated with the indicated concentrations of CFTRinh-172. **F.** Basal (5.5mM glucose) and stimulated (12.5mM glucose) insulin secretory response of MIN6 β-cells incubated with vehicle (DMSO) or 5μM Inh172 (*n* = 3, *p<0.05).

To continue, using primary islets *in vitro*, we sought to validate previous physiological results. First, we corroborated the specificity of CFTRinh-172 on the secretory response using primary islets obtained from *Cftr*^WT^ and *Cftr*^KO^ [*i*.*e*., *Cftr*^*tm1Unc*^ Tg(FABPCFTR)1Jaw/J] mice. As shown in Fig 5A, CFTRinh-172 (2.5–10μM) does not affect insulin secretion in *Cftr*^KO^ islets in response to basal or stimulating glucose, as expected for the concentration range already demonstrated to be specific for *CFTR* [7]. Worthy of note, 5μM of the *CFTR* inhibitor was the lowest concentration to reduce the secretory response to glucose in *Cftr*^WT^ islets. Therefore, we used that concentration of the inhibitor to interrogate *CFTR*-specific secretory responses in the islet. Accordingly, as shown in Fig 5B–5E, 5μM CFTRinh-172 reduced, rather than completely inhibiting, the overall secretory response in mouse, rat and human islets as well as MIN6 β-cells (Fig 5F). Taken together, these results suggest that *CFTR* participates, at least in part, in the secretory response in primary islets.

### *Cftr* is functionally detected in a small proportion of freshly dissociated rat β-cells

**Fig 6:**
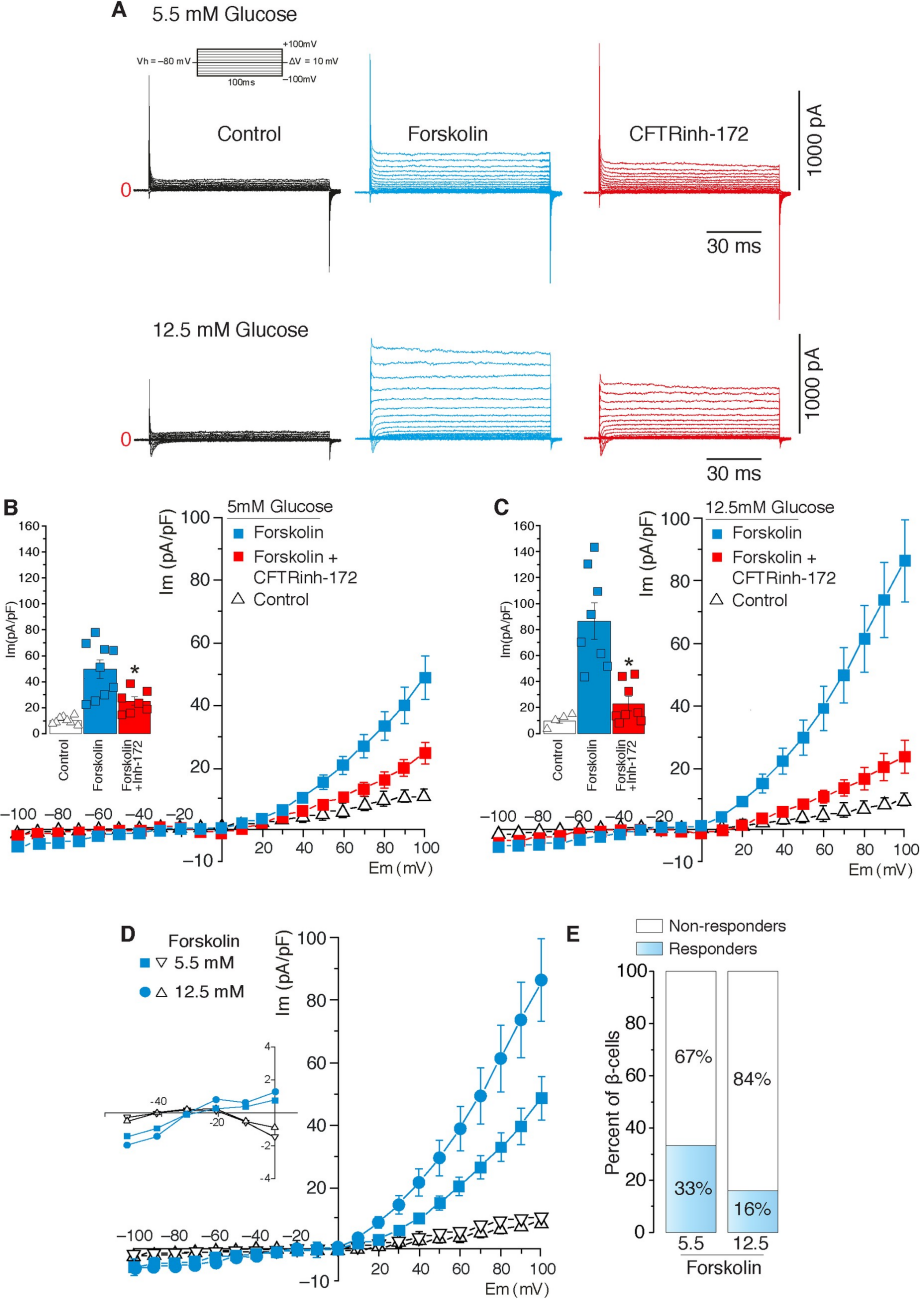
*Cftr* is functionally detected in rat β-cells. **A.** Representative Cl^−^currents recorded using rat primary pancreatic β-cells pre-incubated for 1h in 5.5 or 12.5mM glucose before (*control*, black trace), after forskolin addition (10μM, blue trace) alone or 5 min after adding CFTRinh-172 (5μM, red trace). **B, C.** Current density-voltage relationships of β-cell Cl^−^currents recorded in 5.5mM (*n* = 10) glucose (B) or 12.5mM (*n* = 8) glucose (C) in the presence of forskolin (10μM, blue squares) alone or plus CFTR-inh-172 (5μM, red squares). Insets in B and C denote peak currents at +100mV (*n* = 8, for all conditions, *p<0.05). **D.** Current density-voltage relationship of β-cell Cl^−^currents in 5.5mM or 12.5 mM glucose after addition of forskolin (10μM). Traces show β-cell responders (blue circles/squares, n = 10 and *n* = 8 for 5.5mM and 12.5 mM respectively) and non-responders to forskolin in 5.5mM or 12.5mM glucose (open triangles, *n* = 19 and *n* = 41, respectively). **E.** Numerical proportion of the latter observation.

Previous functional data supports the likelihood that a fraction of the total insulin secretory capacity of human and rodent islets as well as MIN6 β-cells is modulated by *CFTR* (12, 15). To provide further evidence of functional *Cftr* expression, even in a small number of β-cells, channel activity was determined at the single cell level by using the whole-cell patch-clamp configuration on isolated primary rat β-cells. Chloride currents were measured upon imposing a 6-fold asymmetrical Cl^−^concentration difference across the membrane patch, given by [Cl^–^]_o_ = 151.2mM and [Cl^–^]_i_ = 22mM. As shown in Fig 6A, under basal non-insulinotropic or stimulated insulinotropic conditions (5.5mM and 12.5mM glucose, respectively), rat β-cells exhibit a small Cl^−^current that increases in response to forskolin (10μM [33]) and is partially inhibited by CFTRinh-172 (5μM), (Figs 6A–6C and 5A). The corresponding *I/V* relationship plots are shown in Fig 6B and 6C. Note that this conductance became curvilinear and positive as a function of voltage. Indeed, as the voltage increased from negative values to positive ones (becoming >> E_Cl_), Cl^−^increases its probability to move through CFTRinh-172-sensitive channel(s) due to the higher electrochemical potential and therefore, the conductance is predicted to increase asymptotically. An important observation is that CFTRinh-172 blocks *CFTR* with an IC_50_ of 0.3μM and that at 5μM, the inhibitor does not alter Ca^2+^-activated (*Ano1/Ano2*) or volume-regulated Cl^−^currents (VRAC) [7] whereas it has no effect on the secretory response in *Cftr*^KO^ mouse islets (Fig 5A). Remarkably, as shown in Fig 6D and 6E, a relatively low proportion of β-cells revealed Cl^−^currents under basal and stimulated glucose conditions *i*.*e*., ~33% and ~16%, respectively (Fig 6E). The fact that *CFTR* is expressed in a minor and heterogenous subpopulation of β-cells may help explain, at least in part, why others have not been able to detect channel activity by electrophysiological means.

## Discussion

We have provided evidence supporting the thought that *CFTR* is expressed at low levels in a heterogeneous manner in β-cells and that this Cl^−^channel may play a role in the modulation of insulin secretion. To start afresh and to minimize misinterpretations based on previous results, we systematically screened 21 antibodies raised against human and mouse *CFTR* and validated them against pancreatic tissues expressing and lacking this protein (Figs 2 and 3). Six of these antibodies were indeed specific when tested against human and mouse pancreatic tissues (Table 1) but the remainder, were either not sensitive enough to detect low levels of *CFTR* in the islet or produced unexpected staining patterns or high background. For instance, CFF-596, a specific antibody gave the expected staining in the exocrine pancreas, but failed to detect *CFTR* in the islet (S3A Fig), a finding recently corroborated [12], thus suggesting a reduced affinity/sensitivity of the antibody for its antigen. Moreover, in our hands, antibodies CFF-660, -450 and Ab4067 (S3 Fig) did not produce strong staining in pancreatic sections whereas those from the *CFTR* Folding Consortium [34] did, but mainly on the islets (S3E–S3J Fig). Antibodies CFF-432, -570 (S2 Fig), 10B6.2 and MA1-935 were all only suitable for IHC, as shown for CFF-217 and -412 (Figs 1–3 and S1 Fig). Although these results may provide the experimental basis of the general tenet that *CFTR* is absent in the islet, they highlight the need for the use of validated tools to minimize false negative results. In fact, few of the *CFTR* antibodies we tested and validated do support the conclusion that *CFTR* is expressed at low levels and in a heterogeneous way in the pancreatic islet in human (Fig 1A–1C) and mouse (Fig 3A–3E) sections. We consistently detected *CFTR* close to the boundaries of the endocrine cells in human (Fig 1C) and mouse (Fig 3E) islets, confirming the possibility that a small fraction of *CFTR* localizes to the plasma membrane where channel function can be measured. These data agree with epithelial *CFTR* sub-membranous localization, where less than 20% of channels reach the plasma membrane [35], giving the distinctive *"apical"* immunolabeling pattern observed in epithelial tissues.

*In situ* hybridization experiments also demonstrate very low and diverse levels of *CFTR* transcripts in some β-cells, but not in all of them (Fig 1D–1H), complementing previous results suggesting that *CFTR* transcripts are indeed expressed at very low levels in sorted β-cells [11,36,37]. However, it remains to be determined whether these RNA-sequencing studies, that show low expression of *CFTR* reflects β-cell heterogeneity, as recently suggested [12]. In fact, RNA-sequencing data analysis usually fails to provide reliable and accurate gene expression measurements [38,39], in particular related to the expression of genes in heterogeneous populations expressing low levels [40]. This limitation becomes important in pancreatic islets where they comprise subpopulations of β-cells with different genetic programs and functional properties, including the secretory response [22,41]. As previously described, human islets, for instance, contain at least four antigenically distinguishable β-cells with different transcriptomes and secretory responses [42].

*CFTR’s* involvement in the modulation of β-cell electrical activity and insulin secretion has been suggested by several investigators [14,17] but disapproved by others [19]. In fact, *CFTR*, which was originally detected at the protein level in rat α-cells as well as β-cell lines [13], has been functionally implicated in the insulin [14,17] and glucagon [15] secretory responses of human and mouse islets. Our results corroborate specificity for CFTRinh-172 in the secretory response in human, mouse and rat islets (Fig 5A–5E) as well as in MIN6 β-cells (Fig 5F). Importantly, CFTRinh-172 did modulate insulin secretion in WT, but not in islets lacking *Cftr* in a concentration range between 2.5μM and 10μM (Fig 5A), contrasting with the recent suggestion that CFTRinh-172 is not specific [11,18]. Actually, the inhibitor’s lack of specificity appears to reflect its use at or above 10μM [18], as already previously reported [7]. Our results show that specific inhibition of *CFTR* was insufficient to abolish islet insulin secretion in response to glucose (Fig 5A–5E). Similarly, elimination of *VRAC* in mouse β-cells did not eliminate, but rather decreased insulin secretion [43], whereas mouse islets lacking *Cftr* exhibited an apparently normal secretory response [11]. In addition, the reduced plasma membrane depolarization, Ca^2+^ oscillations and insulin secretion [17] or reduced Cl^−^currents and granule exocytosis [14] demonstrated in response to >10μM CFTRinh-172 may suggest that at this concentrations, *CFTR* is not the only CFTRinh-172-sensitive Cl^−^channel involved in the insulin secretory response. In fact, >10μM CFTRinh-172 blocks Ca^+2^- and volume-activated Cl^−^channels [7], regardless of *CFTR* expression [44] whereas ≥20μM also impairs mitochondrial function [45]. Therefore, the inhibition of the insulin secretory response documented in *Cftr*^KO^ ferret islets treated with 20μM CFTRinh-172 [18] could be attributed to off-target effects, including the inhibition of other Cl^−^channels (Ano*1/2/VRAC)* and/or decreased mitochondrial function, both known to alter the insulin secretory response [46,47]. Therefore, the effect of high concentrations of CFTRinh-172 on normal β-cells could be attributed to a combined participation of *CFTR*, *VRAC*, *Ano1* and/or any other potential functional target of the inhibitor, which await identification. Altogether these data suggest that inhibition/elimination of *CFTR* or *VRAC* alone are not sufficient to fully abolish the insulin secretory response and that additional Cl^−^channels are at play, supporting the concept that a multicomponent Cl^−^channel system is present in β-cells, that have the ability to modulate the secretory response [23].

The electrophysiological investigation measuring *CFTR* channel activity in β-cells has been a challenging and daunting task due, at least in part, to the very low and heterogeneous expression of *CFTR* in pancreatic β-cells. Therefore, analyses of an unusual number of β-cells was required to detect CFTRinh-172-sensitive anionic (Cl^–^) currents. We also used a validated whole-cell recording protocol similar to that of Edlund *et al*. [14] instead of the perforated patch strategy used by Hart *et al*. [11] to detect *CFTR* in rat primary β-cells (Fig 6A–6C). These conceptual and methodological considerations may have contributed to the lack of detection of *CFTR* currents in β-cells by other investigators [11]. Recordings show the reversal potential for Cl^−^currents under control conditions or in cells which did not respond to forskolin to be consistent with that predicted by the Nernst equation. In addition, the forskolin-activated Cl^−^current was shifted to the right suggesting additional cAMP-activated currents. Nevertheless, this forskolin-dependent current was partially inhibited by 5uM CFTRinh-172, a concentration that specifically affects *CFTR* currents. The presence of a forskolin-dependent current is also consistent with functional expression of *Cftr* in these cells. However, it is important to emphasize that less than ~30% of β-cells exhibited these Cl^−^currents under basal or stimulated conditions (Fig 6D and 6E), suggesting that the expression pattern of *Cftr* in the islet is heterogeneous and not functionally measurable in all β-cells by electrophysiological means. As briefly mentioned earlier, there is ample evidence supporting β-cell functional variability within the islets [20], where it has been evident that β-cell functional heterogeneity shapes overall islet function under normal and pathological states [21,22]. Therefore, our results demonstrating functional *Cftr* in ~30% of β-cells under basal conditions advocate for a role for this channel in the secretory response in at least ^1^/_3_ of β-cells, a finding that helps mitigate at least partially, some of the disagreements around *CFTR* expression and function in the mammalian islet [11,12,19].

In summary, it remains clinically and mechanistically relevant to validate the hypothesis that *CFTR*, and/or any other Cl^−^channel expressed in β-cells modulate the β-cell secretory response. Demonstrating that *CFTR* is expressed in a subset of β-cells and takes part in their function adds an extra layer of complexity to the pathogenesis of CFRD. Clearly, this, as any other interpretation driven from *CFTR* expression in β-cells, cannot simply discard other mechanisms already proposed as players in the pathophysiology of CFRD. At this point, it is important to note that *CFTR*, irrespective of its low expression and/or independently of its Cl^−^channel activity, may have long-term effects on β-cells physiology or pathology. Further, *CFTR* does not need to be abundant or a Cl^−^channel to play a role in any cell type (*reviewed in* [23]). Therefore, disregarding the possibility that *CFTR* may have a role in β-cell physiology because its Cl^−^channel function is impaired or because its expression is low still remain premature at this point. Although the involvement of β-cell *CFTR* in the appearance of the insidious intermittent hyperglycemia in CFRD remains to be determined, the concept is relevant when considering that the existing relationship between CFRD and exocrine pancreatic insufficiency [48] does not explain the fact that CFRD also occurs in patients without pancreatic insufficiency and that the latter is not a necessary condition to develop diabetes [49]. Together, our observations place *CFTR* as a component of a complex Cl^−^channel machinery in the modulation of insulin secretion and provide an additional piece of information to help explain the impaired insulin secretion that results in CFRD.

## Supporting information

S1 Fig (TIF)

S2 Fig (TIF)

S3 Fig (TIF)

S4 Fig (TIF)
