# Correction: Heterogeneous expression of *CFTR* in insulin-secreting β-cells of the normal human islet

**DOI:** 10.1371/journal.pone.0288417

**Published:** 2023-07-07

**Authors:** Mauricio Di Fulvio, Marika Bogdani, Myrian Velasco, Timothy S. McMillen, Cecilia Ridaura, Lisa Kelly, Mohammed M. Almutairi, Shams Kursan, Abu A. Sajib, Marcia Hiriart, Lydia Aguilar-Bryan

The Supporting Information file legends are incorrect. Please view the correct Supporting Information file legends below.[1]

## Supporting Information

**S1 Fig. CFTR presence and localization in the normal human pancreas.** CFTR immunostaining using CFF-217 (A, C, E, G, I, K and L) of normal pancreas from donors of different ages: 2 months (A-B), 6 months (C-D), 2 months (E-F), 7 months (G-H), 30 years (I-J), and 39 years (K-L). CFTR immunoreactivity is shown in ductal and islet endocrine cells. Shown in B, D, F, H and J are consecutive sections immunolabeled by using the pan-endocrine marker synaptophysin (SYN) to confirm CFTR expression in the pancreatic islets. A larger magnification of the section area in the inset in K is shown in L. Scale bars represent 50μm.

https://doi.org/10.1371/journal.pone.0242749.s001

(TIF)

**S2 Fig. Immunohistochemistry for CFTR.** Normal human pancreas tissues obtained from three donors aged 6 months (A, D, G), 11 months (B, E, H) and 4 years (C, F, i) are immunostained by using anti-human CFTR antibodies CFF-432 (A-C), CFF-412 (D-F) and CFF-570 (G-i). CFTR expression (brown) occurs in both duct (green squares) and islet endocrine cells (red squares). Higher magnifications of the areas noted by green and white inserts i.e., ductal and islet areas, respectively, are shown under each image. Scale bars represent 100μm.

https://doi.org/10.1371/journal.pone.0242749.s002

(TIF)

**S3 Fig. CFTR antibodies not-suitable for immunohistochemistry.** Normal human pancreatic tissues stained with the indicated CFTR antibodies. A. CFF-596 antibody generates non-specific immunostaining in the CFTR-negative control human tissue with the G542X truncation mutation. B-C. CFF-660 (B) and CFF-450 (C) antibodies do not generate specific immunostaining in the positive control ductal cells. D. Antibody Ab4067 does not generate immunostaining signal in the normal human pancreas. E-J. Pancreatic tissue from CftrWT (E-G) and CftrG542X mice (H-J) co-immunolabeled by using the mouse anti CFTR-specific antibody MrPink (J and H), insulin (F and I, Ins) and glucagon (G and J, Glc).

https://doi.org/10.1371/journal.pone.0242749.s003

(TIF)

**S4 Fig. Original blots used in Fig 4**.

https://doi.org/10.1371/journal.pone.0242749.s004

(TIF)

## References

[1] Di Fulvio M, Bogdani M, Velasco M, McMillen TS, Ridaura C, Kelly L, et al. (2020) Heterogeneous expression of *CFTR* in insulin-secreting β-cells of the normal human islet. PLoS ONE 15(12): e0242749. 10.1371/journal.pone.0242749 33264332 PMC7710116

