# Supplementary figures and images for: Heterogeneous expression of CFTR in insulin-secreting β-cells of the normal human islet
Source: PLoS One. 2020 Dec 2;15(12):e0242749. doi: 10.1371/journal.pone.0242749 (PMC7710116; doi:10.1371/journal.pone.0242749)

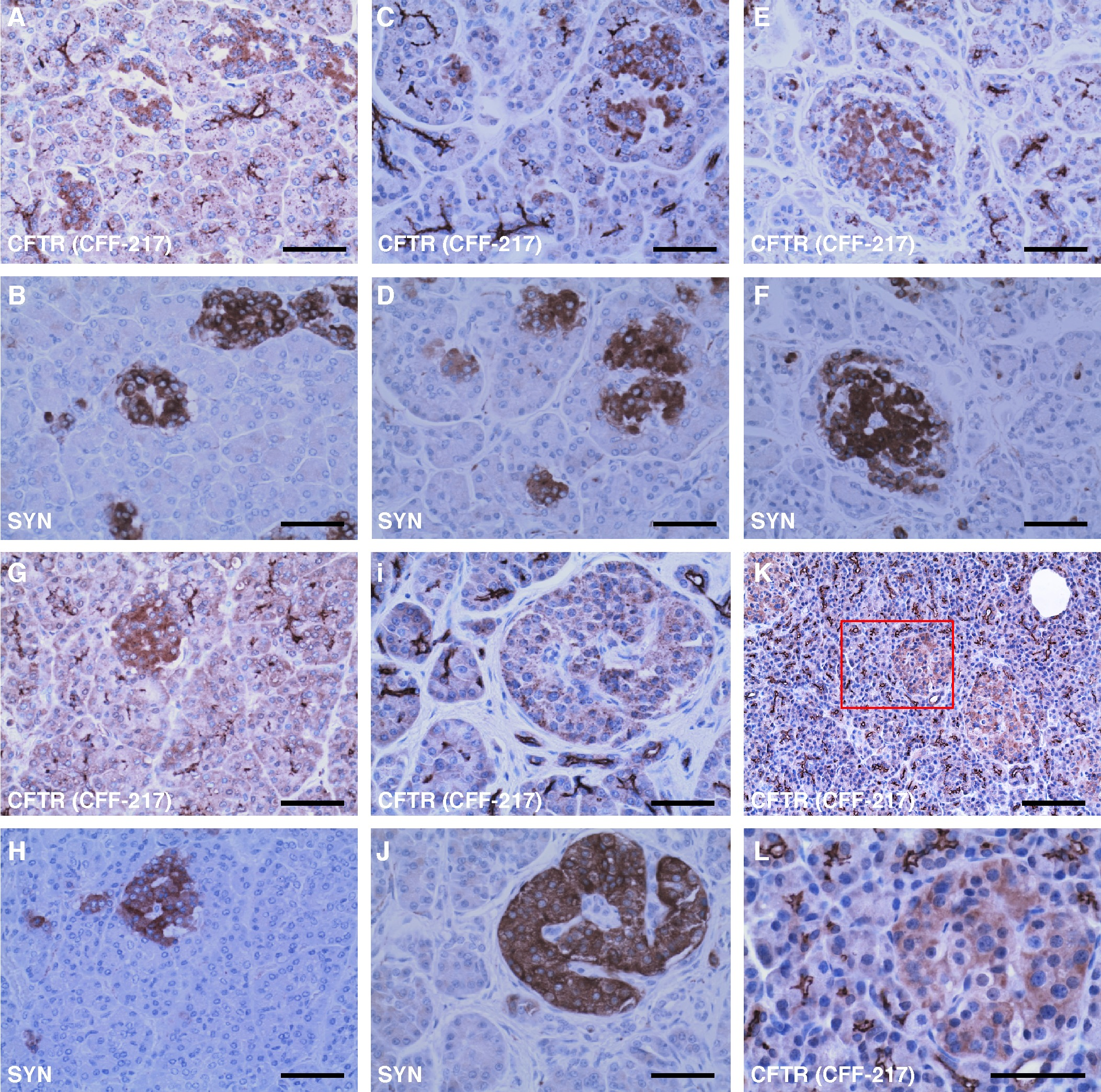

Supplement: S1 Fig — (TIF) [file pone.0242749.s001.tif]

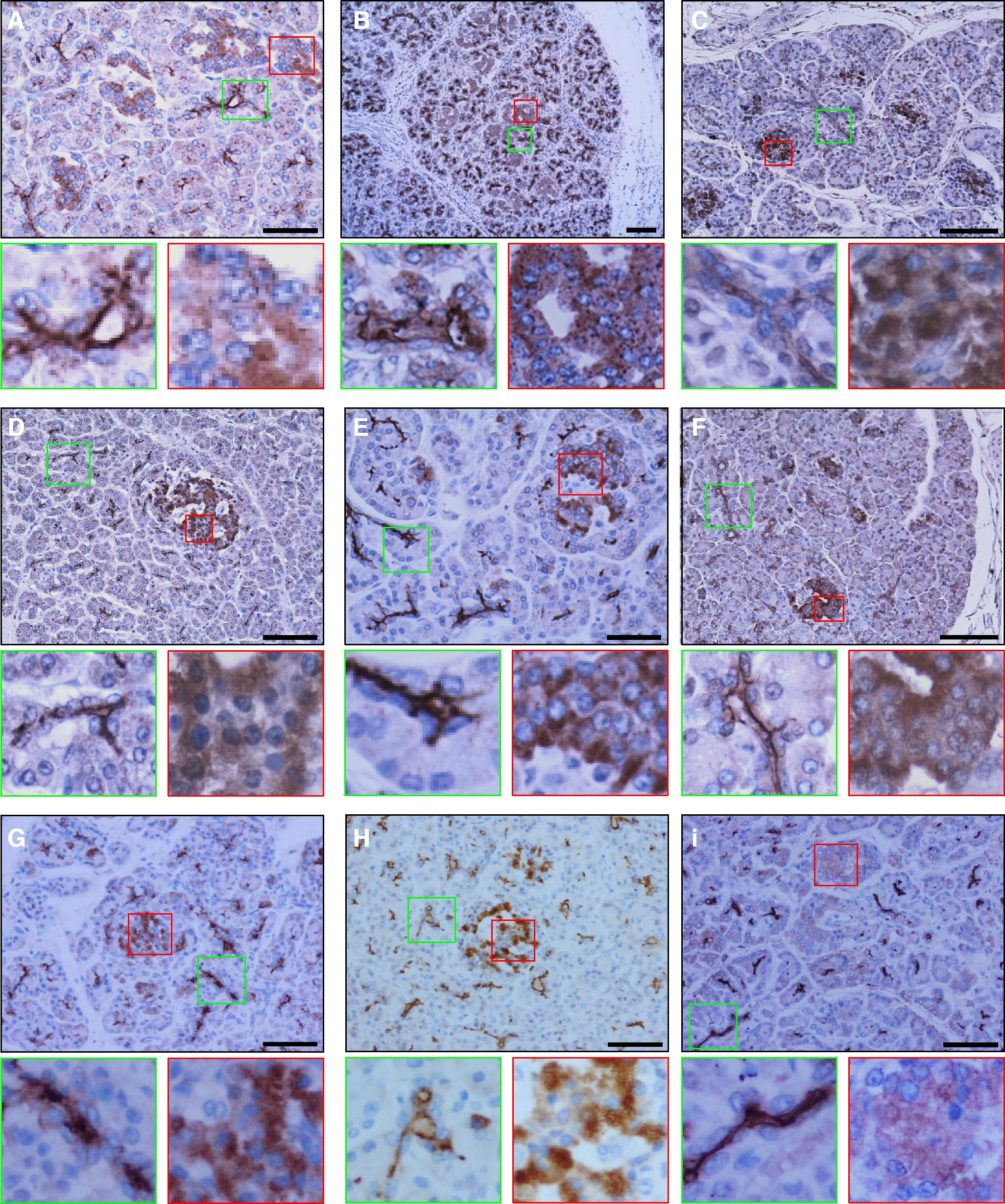

Supplement: S2 Fig — (TIF) [file pone.0242749.s002.tif]

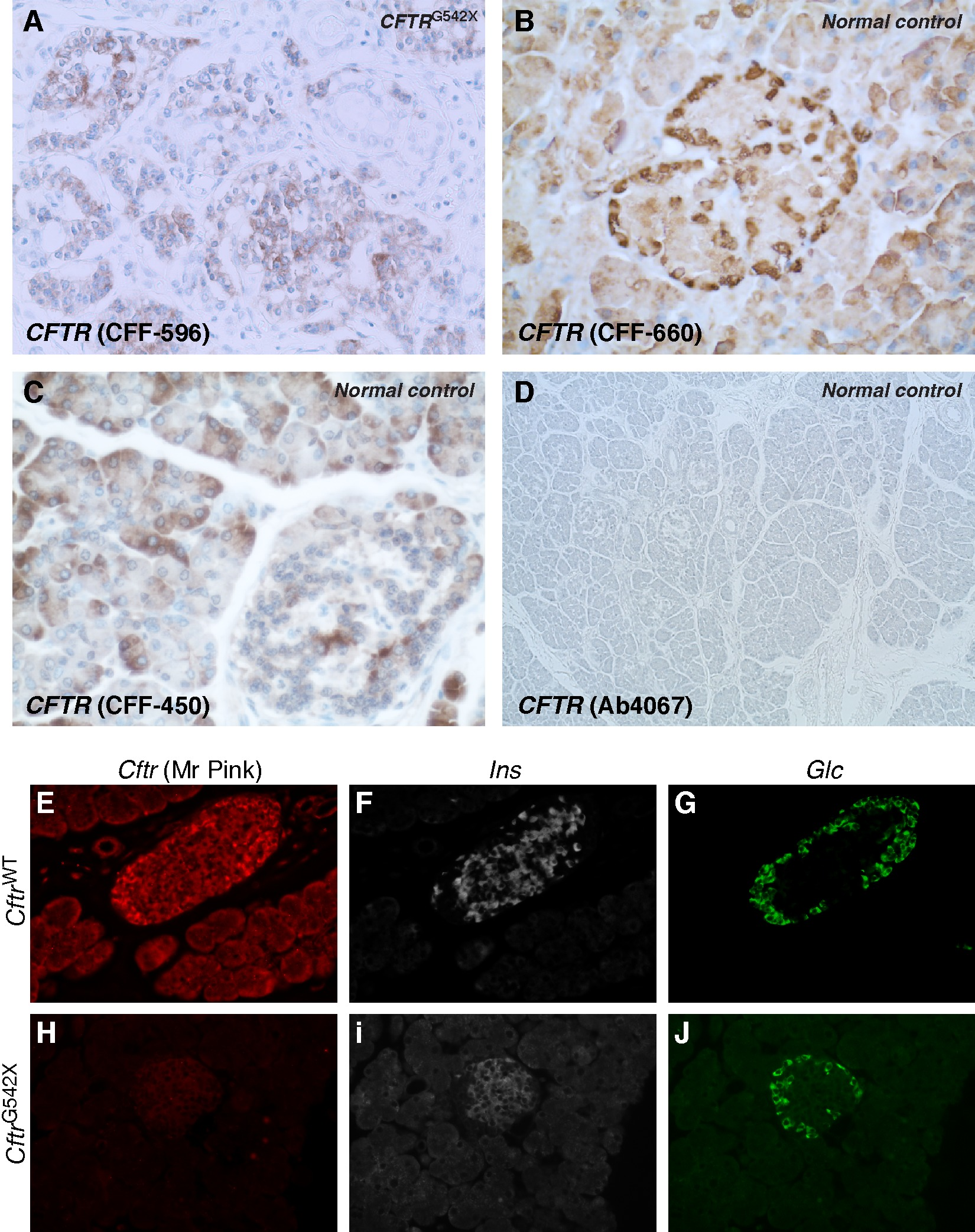

Supplement: S3 Fig — (TIF) [file pone.0242749.s003.tif]

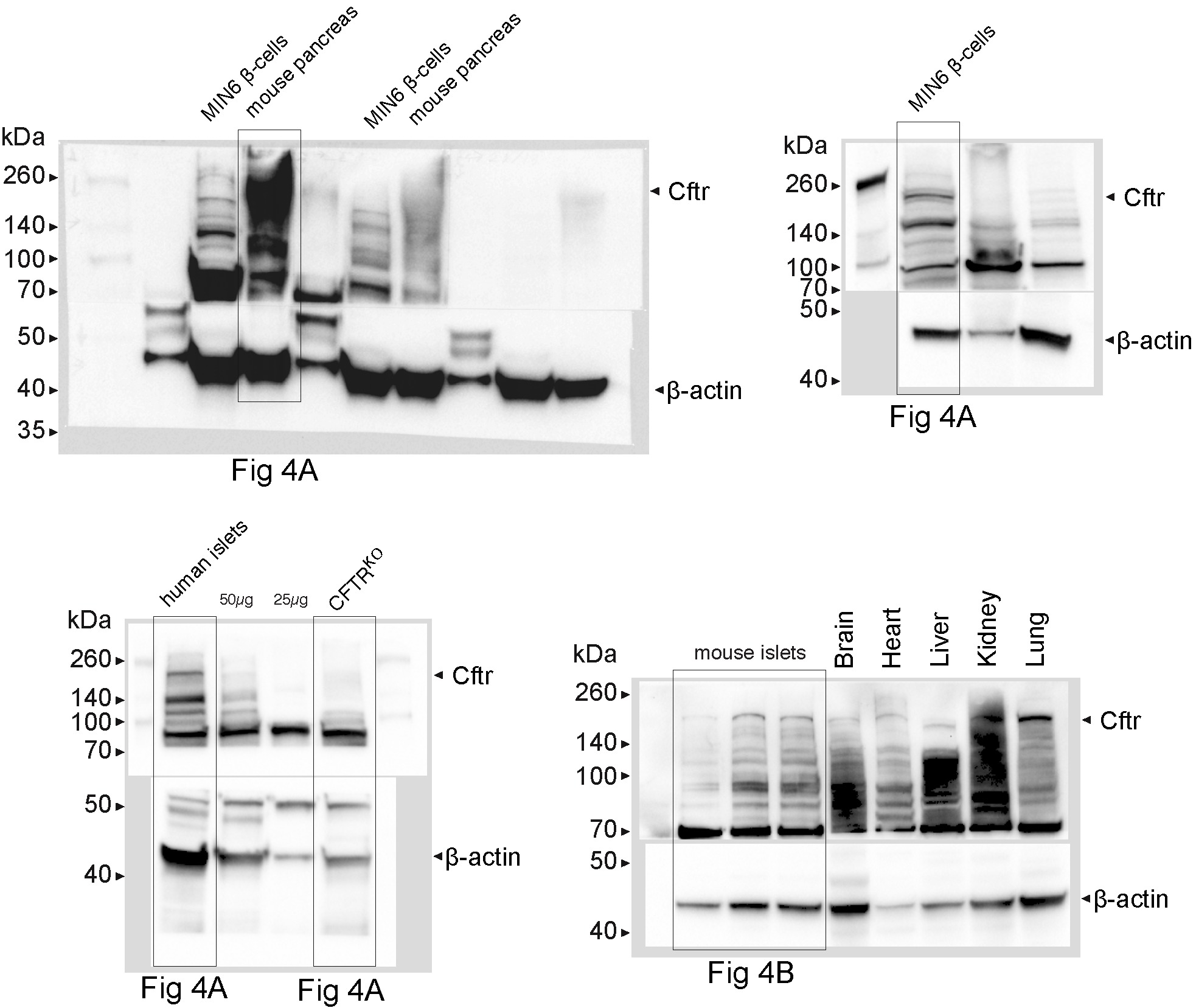

Supplement: S4 Fig — (TIF) [file pone.0242749.s004.tif]
